# Idiopathic sudden sensorineural hearing loss: etiopathogenic aspects

**DOI:** 10.1016/S1808-8694(15)31004-1

**Published:** 2015-10-19

**Authors:** Paulo Roberto Lazarini, Ana Cristina Kfouri Camargo

**Affiliations:** a ENT Professor - Santa Casa de São Paulo, Postgraduate Course Professor - FCM Santa Casa de São Paulo, Professor-Instructor; b Postgraduate student – Otorhinolaryngology – School of Medical Sciences - Santa Casa de São Paulo; c Department of Otorhinolaryngology – School of Medical Sciences - Santa Casa de São Paulo

**Keywords:** hearing loss, idiopathic sudden, sudden sensorineural hearing loss

## Abstract

Several factors have been postulated to elicit the etiology of idiopathic sudden sensorineural hearing loss. Through a bibliographic review, we made a critical analysis of the different etiopathogenic aspects of its clinical manifestation. The most recent studies concerning the possible causes of sudden hearing loss suggest vascular disorders, rupture of the inner ear membrane and autoimmune diseases; however, viral infections have received a great deal of attention in recent years. Little is known about the mechanism of sudden hearing loss. Viruses can cause sudden hearing loss in an acute infection, however the latent form, and its possible reactivation have also been considered as explanations of the cochlear injury mechanism. Even though hearing loss can be explained by a blood viscosity change, experimental and clinical studies do not show any evidence of labyrinthine fibrosis and new bone formation, or labyrinthine membrane breaks. These findings are not in agreement with vascular and rupture membrane factors, respectively. The eventual presence of antibodies against the inner ear suggests that sudden hearing loss pathogenesis may be of autoimmune nature, but the difficulty in establishing the correlation of its morphological and clinical aspects to the hearing loss also do not help to support this statement. Sudden hearing loss is still a controversial and obscure subject in several aspects.

## INTRODUCTION

Sudden hearing loss (SHL) means a sensorineural hearing loss of sudden onset or one that happens in minutes, hours or even in a few days. The hearing impairment varies as far as intensity and sound frequency are concerned, and in general, it is unilateral (98-99% of the cases)^1^, ^2^.

It may be missed in children, specially when it is unilateral. Tinnitus frequently follow the symptoms of idiopathic sudden hearing loss (ISHL) (70% of the cases) and diziness is sometimes present (up to 40% of the cases), thus completing the triad of Ménière. About 10% of dizziness cases may be incapacitating and associated to nausea and vomit. There may also be ear fullness, headache and viral infection symptoms of the upper airways^1^, ^2^, ^3^.

One of the few emergencies in otology, the ISHL affects mostly those in their fourth decade of life, involving both the right and the left ears in equal proportions, and the same trend is seen genderwise^4^.

It affects 5 to 20 persons for each 100 thousand individuals. It is estimated that there are approximately 4 thousand new cases per year in the United States, and 15 thousand all over the world^7^.

In Brazil we lack good epidemiological references as to the true incidence of SHL, due to the difficulty in safely assessing its incidence. Poor cultural and socioeconomical conditions, together with the possibility of spontaneous recovery before seeking medical help or the very disregard towards light symptoms are responsible for this epidemiological picture^3^.

Although no seasonal influence has been proven for ISHL, it is known that patients with such disorder are mainly affected after an upper airway infection or herpes labial infection^8^.

The hearing loss (HL) caused by ISHL may become permanent or regress either totally or partially. Many authors report that spontaneous recovery happens in 45 to 60% of the patients^4^, ^6^, ^9^, ^10^. In a more detailed fashion, it is estimated that about 25% of patients have total spontaneous recovery; 50%, partial and 25% present no recovery at all^2^.

Since ISHL physiopathology is still unclear, there are disagreements as to its true cause^11^, ^12^. Such fact becomes even more evident when it happens as an isolate clinical situation, that is, symptoms restricted to the inner ear only. Thus, there are many theories as to the origin of this disease, including viral infections, blood disorders, immune disorders and perilymphatic fistulas.

Having stated that, our goal is to assess ISHL as to its main and possible etiology and physiopathology.

## BACKGROUND

Prosper Ménière was probably the first author to clearly describe a case of SHL without a definitive cause^4^, ^5^, ^6^, ^13^, in 1861; however, it was DeKlein, in 1944, the first to report a clinical study with 21 patients with SHL, listing causes of hearing loss among inner ear hemorrhage, acute and chronic inflammations, traumatic fractures, multiple sclerosis, brain tumors, ototoxic drugs, emboli, hypercoagulability, radiation, pregnancy and herpes zoster as potential predisposing causes.

Later, Rasmussen in 1949, apud Fowler (1950) and Hallberg (1956), report cases of ISHL and discuss the disease origin, proposing the vascular origin and the neuritis of the VIII nerve as possible causes. Following that, Lindsay & Zuidema (1950) also studied patients with SHL and discuss possible causes. According to these authors, vascular diseases such as thrombosis could justify the hearing loss, however the age range did not match; hemorrhage would be another possibility, provided the patients had previous history of coagulation problems or head injury. They then consider, although hypothetic, the possibility of vasospasms, besides infection as the possible cause for hearing loss (HL), what could then justify this variable pattern of auditory and vestibular involvement.

Back in 1950, Fowler present some SHL cases and stresses that most of the patients had a psychosomatic disorder associated. He reports not only the importance of the supratentorial disorder, but also the change in blood viscosity, as possible causes for SHL. It was also in 1950 that Opheim, apud Hallberg (1956), reported cases of SHL, highlighting the possibility of an acute increase in labyrinth pressure as being responsible for such happening.

Later, in 1952, Moulonguet and Bouche, apud Hallberg (1956), reported the case of a young physician with SHL after having used an intravenous medication, and also considered that the arteriolar spasm caused by this drug caused the clinical signs and symptoms in this patient.

Disorders of the labyrinthine circulation such as the occlusion of microvessels in the inner ear and cochlear hemorrhage have been considered as possible causes of ISHL, specially because they are of sudden onset, notwithstanding, there are a number of non-compatible aspects, such as age range, risk group and prognosis^2^, ^9^, ^14^.

It may be that an alteration in blood viscosity caused by a prior disease be consider a predisposing factor, however, not as a primary cause of sudden hearing loss^13^.

In 1957, Van Dishoeck & Bierman^15^ influenced by previous studies and seeing that flue-like symptoms almost always precede ISHL, are among the first to report a viral infection as a possible cause for such disorder, specially when they compared cochlear lesions found in temporal bones of individuals with ISHL with those with viral labyrinthitis.

Heller & Lindenberg^16^ in 1955, and Lindsay & Zuidema (1950) had also published a study in which they identified some patients with hearing loss possibly caused by an infection by the varicella-zoster virus and by the measles virus; however, it was the retrospective study carried out by Hallberg (1956) who had the first sample big enough to try and establish any relationship between hearing damage and clinical findings. Then a new discussion took place for otologists: what were the possible causes of ISHL?

Autoimmune causes are included as possible causes for ISHL. In 1979, McCabe was the first to associate SHL and progressive sensorineural hearing impairment with autoimmune diseases and to consider it because it can be treated. Not always identified, diseases such as rheumatoid arthritis, lupus, nodose polyarthritis and others are possible mediators of the antibody and immunocomplex processes that may be involved with the origin of the sudden hearing loss^17^, ^18^, ^19^.

Perilymphatic fistulas, in some cases may also explain the hearing loss and its spontaneous recovery in patients with ISHL^14^. However, the lack of histologic, post mortem evidences in temporal bones of patients who had SHL at some point of their lives, because of a possible rupture of the labyrinthine membrane contradicts the former considerations^14^.

## MATERIALS AND METHODS

This study was carried out with a survey of the different scientific papers related to the theme, with a critical and comparative analysis of these papers.

## DISCUSSION

A cause of great expectation, ISHL is a terrible experience for the patient. The sudden silence, followed, sometimes, by tinnitus and/or vertigo, represents, not only the loss of inner ear function, but also the impairment to the patient's psychological status. Although not very relevant, it is an otologic emergency, which bears no established cause or physiopathology and, therefore, without established treatment. Thus, it is a problem that places the physician in a difficult clinical situation. As to clinical evolution and hearing recovery, they are both question marks in the course of the disease.

The definition of ISHL is not universal. It is a sensorineural hearing loss that may vary as to the concept of the word “sudden”. There are cases in which the hearing loss may be sudden or happen in a short period o time until it becomes complete^4^, ^20^. Others report that this hearing loss may develop along a few days^1^, ^2^, although many authors accept the time period of up to 72 hours^9^, ^15^, ^21^, ^22^. In a shorter and more contemporary way, it may be defined as a 30dB hearing loss minimum, in at least three continuous frequencies of up to 72 hours of onset. It varies according to intensity and frequency affected; and in order to be classified as ISHL all known causes have to be ruled out previoulsly^9^, ^15^, ^21^, ^22^.

The etiology of the ISHL, as the very name states (idiopathic), is still unknown, what makes the topic still very controversial. In only 10% of the cases the cause can be found^9^, ^21^, ^23^, according to Nakamura et al. (2001), over 45% of ISHL cases bear unknown causes. Vascular causes also compete with autoimmune diseases and the rupture of the labyrinthine membrane, and also viral infections^18^, ^21^, there still are psychosomatic disorders^5^, ^24^.

The emotional factor as possible cause of ISHL may be considered as long as all the other physical causes are ruled out. An emotional disorder may alter the neurovascular mechanism due to an ANS response. There would be vascular contractions, viscosity alterations and eventual congestion, anoxia, increase in capillary permeability and local metabolic impairment, what would justify a SHL^24^. Still, estrogen and adrenergic disorders generated by emotional disorders may also cause changes in blood viscosity, causing and thus being responsible for SHL. It is believed that such happening is only possible if there are anatomical variations to the terminal cochlear vessels which predispose an obstruction caused by this increase in blood viscosity and consequent anoxia. Actually, it is more likely that a combination of emotional and organic factors be one of the possible origins of ISHLI^5^.

The intense anguish and restlessness during consultation and the great variability in results are factors that suggest a psychosomatic SHL^5^. Some other signs may also serve as diagnostic hints: a) Severe bilateral hearing loss; b) saucer-shaped curve in the audiometry in 80% of the cases; c) unexplainable discrepancy among tonal and vocal audiometric tests; d)

Noteworthy and disproportional recovery after clinical treatment.

### Vascular Causes

The fact that the cochlea is mainly supplied by one single artery, the labyrinthine artery, and this is a terminal artery, makes the inner ear very much prone to circulatory alterations^25^. Thus, the vascular hypothesis is plausible. Notwithstanding, if the SHL were caused by a vascular disease, it would be reasonable to believe that the risk factors and the age range for both would be similar, however, they are not.

In vascular causes, the problems may be present on the blood vessel wall, as is the case in arteritis and spasms, or intravascular, such as gaseous emboli, fat emboli, polycythemia, hyperviscosity (Waldenströn macrobulinemia), sickle cell crisis, and others. Cochlear damage would be secondary to anoxia or hypoxia generated and could occur due to three different mechanisms^14^. They are:

a) Total and permanent vessel occlusion;
b) Total and temporary vessel occlusion;
c) Cochlear blood hypo flow.

Total and permanent occlusion of a blood vessel would cause anoxia, and consequently necrosis of labyrinthine membranes, fibrosis and ossification of the inner ear. This would justify the hearing loss, but not the recovery of tonal thresholds. Moreover, rare are the times in which fibrous and bone proliferation were found in some study. Thus, this is not a very probable theory that could justify the ISHL pathogenesis.

Cochlear tolerance to schemia is very limited and the action potential starts to be impaired after 60 seconds of anoxia^6^. When there is total circulation blockage, but temporary, after 30 minutes the hair cells, the ganglionic cells and the spiral ligament are already affected, besides, there are neuronal loss and small alteration in the tectorial membrane. When blood flow goes back to normal, the action potentials are already irreversibly impaired. After one hour of vessel obstruction, there is no more cochlear function recovery, and after six months there is an invasion of fibrous and bony tissue to the inner ear spaces. This mechanism also explains HL and even, depending on how long the vascular occlusion lasts, the recovery of tonal thresholds; however histopathological findings are still uncommon in the temporal bones of the SHL cases assessed. Therefore, this is also an unlikely hypothesis to explain ISHL physiopathology.

Low blood flow, the third mechanism involved, happens because of a hyperviscosity, generating a drop in oxygenation (insufficient to maintain cochlear metabolism) and cochlear hypo function. This would be the most acceptable theory among vascular causes, since it does not only justifies HL, but also the possible audiological recovery and even cochlear histological findings^2^, ^9^, ^14^.

Blood flow is inversely proportional to blood viscosity. Viscosity depends on the hematocrit, serum viscosity, red cell aggregation and RCD. RCD is defined as a physicochemical characteristic that allows the red blood cell to pass through capillaries with diameters that vary between 3 and 12µm, although in average their diameter is always above 7µm. It is believed that a reduction in RCD may cause some cochlear injuries and be related to SHL^13^.

The hematocrit plays a more important role in the blood flow of the great vessels, while RCD plays an important role in the circulation blood flow, specially in smaller vessels, and it may be affected under some situations such as coronary artery diseases, blood diseases and cellular shape changes, renal disease, post-operative alterations and post AMI alterations, serum protein alteration, peripheral vascular disease or diabetes mellitus. It is also thought that smoking may have influence over RCD^13^.

It is believed that RCD reduction may be either an acute or chronic phenomenon, and it bears great incidence in cases of ISHL. A schemia secondary to RCD reduction could be clinically mistaken with an obstructive vascular cause and explain the hearing loss, and moreover a possible recovery of tonal thresholds in some cases of ISHL. Hence the use of drugs that could enhance blood viscosity and RCD would be justifiable.

Still, we know that an acute upper airway infection, together with a viremia, alters RCD and can lead to an ischemia on the vascular stria due to a low blood flow and increase in arterial-venous shunt; both the ischemia and acidosis generated worsen RCD reduction, thus causing a vicious cycle. Thus, if an upper airway acute infection reduces RCD and is associated to a viremia, the ISHL pathogenesis may associate vascular and viral factors as ISHL origin^13^. The virus may attach the red cells and cause hemoagglutination, causing a state of hypercoagulability, with a high consumption of prothrombine in some cases and, consequently, temporary vascular occlusion^6^. An emotional disorder may also trigger alterations in blood viscosity and thus participate in the ISHL etiopathogenesis^24^. When such alteration happens in a transitory manner, the HL, vertigo and tinnitus are short-lived, as in a Ménière crisis; if definitive it causes irreversible HL.

Allergic factors, despite rare, may also cause changes in the cochlear microcirculation or, by anaphylactic phenomena, and trigger a change in capillary permeability. Therefore, the physiopathology in these cases is really more related to a vascular alteration then the allergic cause itself^3^.

The fact of the matter is that SHL under blood hyperviscosity is highly controversial. Being that because of a reduction in blood flow in the capillaries that nurture the stria vascularis and consequently cause ischemia in the inner ear, or a reduction in the oxygen transporting capacity because of a slower blood flow and consequent generation of micro thrombi and lesion to the cochlear endothelium, thrombosis to the venous system of the inner ear; or any other hypothesis, it is clear that there is no absolute way to prove^25^. It is known, however, that all the clinical manifestations of the hyperviscosity syndrome respond to plasmapheresis.

The inner ear hemorrhage could also be an important explanation for ISHL. Secondary to the use of anticoagulants or diseases that cause low platelet levels, such as leukemia and aplastic anemia, traumas, meningitis, subarachnoid hemorrhage and adenocarcinoma metastasis, this bleeding would then cause a sudden pressure rise in the endolymph or perilymph, biochemical and osmolarity alterations in the intracochlear fluids, intracochlear conduction disorders and ischemia of the peripheral region next to the bleeding site. Histological alterations found in the cochlear of these thrombocytopenic patients show and prove such bleeding in the peri and endolymphatic spaces, besides cellular infiltrate. Notwithstanding, considering that the perilymphatic space hemorrhage is responsible for SHL, the mechanisms are not as clear as they seem, since many arguments contradict them. We did not notice any blood-induced hearing loss in the perilymph during stapedectomy; no histology study show damage to the cochlear structures caused by the presence of blood in the perilymphatic space after stapedectomy; and even after the experimental infusion of blood in the swine perilymph; no potential amplitude or threshold alteration was noticed on the electrocochleogram. Therefore, not even an insidious hemorrhage in the perilymphatic space could cause SHL: thus bringing about a huge stalemate. Despite a not very clear physiopathology, there seems to be an intimate relation between the aforementioned diseases and the hearing loss^26^, having seen that in many MRI tests such finding (inner ear bleeding) is highly present.

Still, among vascular causes, it is known that western diets offer a greater risk of ISHL when compared to the Japanese diet, since ours is more loaded with saturated fat. Western diet causes higher platelet aggregation and factor VII activity, increasing blood coagulability and the risks of vascular obstruction. Now, the eastern diet, richer in vegetal oils, has more important antithrombotic and antiatherosclerotic properties that prevent vascular diseases^27^.

Thus, a history of vascular disease or platelet or red blood cell alterations are the only clinical findings that could lead us to infer a vascular cause for ISHL; Histopathologically, we should find fibrous and bony proliferation in the cochlear and semicircular canal spaces, or at least, the hemorrhage in the cochlear spaces, proven by not only image studies, but also by post mortem^14^ tomographic scans. Other histological findings could exist, such as Corti organ, stria vascularis, spiral ligament and cochlear neurons degeneration: it is likely that such differences are due to the degree of vascular involvement or differences in the pathogenic mechanism^28^.

### Autoimmune causes

Because of the presence of antibodies against antigens in the inner ear and the formation of immune complexes in the stria vascularis, endolymphatic sac and duct, an autoimmune disease may be consider a cause that favors sudden or progressive HL^17^, ^18^, ^19^, ^23^. McCabe (1979) introduced the concept of SNHL of autoimmune origin as a nosologic entity, however, because of the difficulty of having anatomo-pathologic documentation of the autoimmune disease among the possible causes of SHL, is one of the most controversial issues in the literature^29^.

Autoimmune SHL may happen alone – specific organ-disease – as in the thyroiditis of Hashimoto, or associated to one systemic disease – non-organ-specific disease, such as Cogan's syndrome, nodose polyarthritis, systemic lupus eritematous, rheumatoid arthritis, Berget's disease, Sjögren's syndrome, Behcet's syndrome, ulcerative colitis, rheumatoid arthritis, and others^3^, ^23^, ^29^, ^30^. The cochlear tissue in itself may be the target of the autoimmune reaction, but maybe because of a lack of membranous labyrinthine tissue, the triggering of the self-aggression mechanism may be less frequent in the isolate form of the disease when compared to its systemic counterpart.

The immune reaction in the inner ear caused by the endolymphatic via is more intense than that in the middle ear. It is characterized by cell infiltration (initially by macrophages, followed by T-helper lymphocytes) and, after three weeks, T suppressor cells coming from the blood circulation. There still is an inflammatory reaction with increase in local antibodies and intracochlear fluids, with consequent damage to the inner ear (degeneration of the spiral ganglion and stria vascularis, Corti organ and cochlear duct atrophies), besides an intimate involvement of the endolymphatic sac: if destroyed it reduces the immunogenic response. During the secondary response, the endothelial cells of the spiral modiolus vein are activated, thus taking on the characteristic of a differentiated endothelial vessel, and recruit lymphocytes of the blood circulation^4^. In 1999, Alvarenga et al. noticed that, although audiologic symptoms prevail in patients with systemic autoimmune disease, the same is not true with audiometric alterations. Thus, it is suggested the possible existence of an additional protection factor in the inner ear that is not present in the other organs susceptible to autoimmune diseases. Not only the smaller antigenic mass, but also its lower vascularization, non-fenestrated cochlear capillaries and late embrionary development may explain this lesser involvement.

Although the labyrinth immunity be similar to that of the brain (blood-labyrinth barrier separates the labyrinth from the blood circulation, keeping the ionic characteristics within the cochlear environment), the inner ear immunity seems to be more immunoresponsive, because the antigens present in the cochlear quickly stimulates the systemic immunity^29^. This may be an explanation that justifies, despite the small antigenic mass and the vascularization, the possibility of an autoimmune disorder causing ISHL.

Sometimes, immune responses may happen not directly related to an autoimmune disorder or antigen, but rather due to the use of a drug or antibody.

Toubi et al. (1994) found a growing prevalence of anticardiolipine antigen (aCL) in patients with ISHL, without any association with autoimmune disorders. It is known that this antibody bears important association with vascular thrombosis and is not always correlated to clinical alterations; its presence may be secondary to some viral infections and it probably justifies the hearing loss, although we need more studies, based on larger samples, to confirm it. Thus, SHL cause would not be purely caused by an autoimmune alteration, but rather triggered by a viral infection or vascular disorder. This is another factor against autoimmune disorders causing ISHL.

Apha-interferon (a-IFN), on its turn, is a drug used to treat acute and chronic viral infections, neoplasias and autoimmune disorders, and it generates side effects such as psychiatric disorders, blood alterations, autoimmune disorders (thyroid diseases, type I diabetes mellitus and, more rarely, hearing loss. This drug may cause tinnitus and hearing loss, however, if discontinued, in 7 to 14 days the symptoms disappear. This all happens because endothelial anticellular antibodies appear and cause microvascular lesions, thus justifying the clinical picture^17^. It is also noticed that in these cases, the HL is not directly generated by an autoimmune problem, but rather by the use of a drug and consequent vascular alteration, thus ratifying the importance of a vascular disorder in the ISHL origin.

Histologic alterations in cases of SHL due to autoimmune disorders are probably associated, in reality, to vasculitis and its consequences to the organ of Corti and the neurons^3^. Some studies also show^28^ the presence of labyrinth fibrosis and cochlear ossification in autoimmune HL: as in ISHL this histologic finding is not commonly found, this is one of the reasons why this theory is still considered not very likely. We may find retrograde neuronal degeneration, endolymphatic edema; atrophy of the endolymphatic duct, spiral ganglion, organ of Corti and stria vascularis and, even macrophages and endolymph precipitates^23^. Thus, the physiopathologic mechanism of the ISHL and its autoimmune origin is not yet clear^23^, ^28^, ^29^, and there are many factors that contradict this theory. Despite the fact that some microscopic characteristics are similar, as previously said, both fibrosis and ossification in the labyrinth that may be present at autoimmune SHL are different from the ISHL histology findings, and further reinforce the vascular cause theory. Moreover, autoimmune HL is bilateral and insidious, it may happen in weeks or months, and it is usually fluctuating and asymetrical^31^. It affects both membranous labyrinths, simultaneously or not and it mainly affects, at a later stage, the high frequencies. Still, autoimmune HL affects mainly females (such prevalence also exists in systemic autoimmune diseases), and this does not agree with the ISHL epidemiology^29^. However, it is important to highlight that despite ISHL, as previously mentioned, is rarely bilateral and fluctuating, such symptoms do not rule out autoimmune origin.

A number of hematological exams are carried out in order to try to establish the diagnosis. Notwithstanding, such proof may not present answers in cases of localized autoimmune processes, because rarely serum levels to make exams positive are reached. Frequently, the diagnosis of autoimmune hearing ends up being based on clinical history, without chance of in vivo histopathological confirmation^30^.

### Membrane Rupture

As to membrane ruptures, in studies carried out by Simmons (published in 1968 and 1979), they submitted that the rupture of one of the labyrinthine windows (oval and round) would cause the loss of perilymph and consequent pressure alteration in the relationship between compartments with perilymph and endolymph. In 1986, Kohut et al. and Kamerer et al. in 1987, apud Yoon et al. (1990), described the presence of a labyrinthine fistula causing micro fissures between the niche of the round window and the posterior semicircular canal ampule. With that, there would be a rupture of the Reissner membrane and the fluid mixture, with consequent cochlear hypo function, but not a broad cochlear dysfunction^14^.

Moreover, no rupture of the cochlear windows, or either Reissner or basilar membrane rupture was seen in any of the studies carried out by Schuknecht & Donovan (1986) and Yoon et al. (1990) that would justify such theory.

It is important to bear in mind that such occurrence usually happens after sudden pressure alterations in the middle ear, as are the cases of head injuries, barotraumas, stapedectomies or intense physical exercises and not spontaneously^3^, ^28^.

### Viral Causes

The viral infection, due to a number and considerable circumstantial evidences is currently considered as one of the major causes of ISHL. The broad association with the histological findings on temporal bone, seroconversions of specific viral titles, and in a lesser degree, but not less important, connection with prior history of a flue or another prior viral disease is proof of such fact.

Considering the virus as a possible cause, this suggests that the agent responsible has a broad distribution in the human body, besides affinity for the nervous tissue. Either the acute or the latent attack may then generate damage to the inner ear and consequent hearing loss^2^. Such compromise may happen in intra-uterine life, in childhood, adolescence or adult age, without the occurrence of any histology distinction^3^.

Upper airway infections (UAI) and intracranial infections may reach the inner ear through the blood (stria vascularis) and through the cochlear aqueduct or internal acoustic meatus IAM^3^, ^32^. Notwithstanding, it is noticed that the few cases of meningoencephalitis have HL associated, because the cochlear aqueduct present defense mechanisms that prevent the spread of the viral agent for the inner ear, reducing the risk of structural lesion and consequent hearing loss^32^.

Although acute viral infections may cause such damage, latent infections and their reactivation may also explain the lesion. The main latent viruses are part of the group of herpes virus: they are ubiquitous, bear strong neurotropism as characteristic; they not always cause symptoms (subclinical infection) and have complex relation with ISHL. They are herpes simplex, herpes simplex^2^, varicella-zoster (VVZ), cytomegalovirus and the Epstein-Barr.

Other agents are known causes of hearing damage also in fetuses and newborn babies, such as the ones from syphilis, toxoplasmosis and rubella - STORCH (syphilis/ toxoplasmosis/ rubella/ CMV and herpes virus)^33^.

Almost all the SHL cases caused by virus are unilateral. The bilateral occurrence is rare. Depending on the viral agent, the hearing loss may present a certain pattern. For example, mumps courses with severe and irreversible HL, while HL caused by VVZ is of low severity and reversible^34^.

Due to the cochlear arterial blood supply and venous drainage, there should be a correlation between cell degenerations^2^. However, damage happens initially on the cochlear endolymphatic compartment structures and the degree of hair cell atrophy is progressive, starting with mild changes in the upper turn up to complete loss in the basal turn^15^. Later, the ganglionic cells degenerations is greater and that of hair cells is minor. Differently from that, in the case of a complete arterial obstruction, the degeneration of ganglionic cells, stria vascularis, spiral ligament and other inner ear structures is more diffuse; after some months, the ear is subject to an invasion of fibrous tissue and, sooner or later, cochlear ossification^15^.

The lack of correspondence between the structural degenerations (ganglionic cells are more affected on the apical turn and hair cells together with dendrites on the basal turn) thus suggest that the ganglionic cells of the apical turn are the primary target of the causal factor. Therefore, an isolated lesion on the ganglionic cells of the apical turn without loss of corresponding sensorial structures is not compatible with the theory of vascular ischemia as a cause for SHL, and much less with membrane rupture, but it is rather compatible with the viral origin.

Despite the fact that cochlear vascularization is equivalent in normal ears and ears with ISHL, it is noticed that there are more histopathological alterations on cochleas affected by ISHL than in normal cochleas, or even on those affected by presbicusis^2^.

However, although we see many similarities as to anatomopathological alterations among the ears affected by ISHL and ears affected by viral infections, some evidences are still contradictory as to the fact that the virus may be a causal factor of this clinical manifestation.

It is not always possible to establish a direct causal relation with the virus, because the antibody titles may not be significantly altered in a SHL supposedly caused by a viral agent^35^, may be due to the fact that it may be a latent and reactivated infection. Moreover, even if some study is able to demonstrate that a certain virus or bacteria has infected the patient, it is not possible to prove that this agent is responsible, at that moment, for the structural lesion in the inner ear^21^.

Moreover, the levels of MxA, an a-IFN protein marker of systemic viral infection in leucocytes no always present significant increase in patients with ISHL^12^. Some clinical counterpoints also collaborate to make it difficult a direct cause and effect association between viruses and ISHL. For mumps, for example, age associated symptoms and prognosis all challenge this correlation^36^. While parotiditis usually affect children bellow ten years of age, it has an irregular incidence of symptoms associated to tinnitus, vertigo and bad prognosis, SHL is more common between 30 and 50 years of age, and about 70% show hearing improvement.

Even then, the fact that the structural lesions found in temporal bones of ISHL patients be very characteristic and similar to the ones found in temporal bones affected by a viral infection contribute to this theory (viral cause) as the main etiology for ISHL^1^, ^2^, ^9^, ^11^, ^16^, ^37^. The major lesions happen to the organ of Corti, the ganglionic cells, the nervous fibers (reduction in number), tectorial membrane and, less, to the stria vascularis^2^, ^14^.

## FINAL REMARKS

Having said that, we notice that autoimmune diseases, and specially membrane rupture, seem not to have important correlation with idiopathic sudden hearing loss. Vascular disorders and viral agents, however, are the causing factors most likely to justify this hearing loss, although a prior vascular disease or viral exposure is not necessarily inherent to the origin of the HL. ISHL low incidence, the difficult access to the inner ear for better studies on unknown diagnosis and physiopathology make it difficult to achieve better conclusions as to the very origin of this clinical manifestation.
